# A Rare Cause of Angina After Coronary Bypass Grafting; Left İnternal Mammary Artery to Pulmonary Artery Fistula and Successful Treatment with Transcatheter Coil Embolization

**DOI:** 10.5935/abc.20190196

**Published:** 2019-11

**Authors:** Ali Nazmi Calik, Can Yücel Karabay, Evliya Akdeniz, Yiğit Çanga, Baris Gungor, Omer Kozan

**Affiliations:** 1 Doktor Siyami Ersek Gogus Kalp ve Damar Cerrahisi Egitim ve Arastirma Hastanesi, Istanbul - Turkey

**Keywords:** Mammary Arteries/surgery, Pulmonary Artery, Arterio-Arterial Fistula, Embolization, Therapeutic, Cardiac Catheterization, Drug-Eluting Stents, Self Expandable Metallic Stents

## Abstract

Fistula from left internal mammary artery (LIMA) to pulmonary artery (PA) is rarely encountered in daily practice. In recent years, endovascular therapy options have emerged for the treatment of fistula formations and replaced with surgery.

A 53-year-old man admitted to our outpatient clinic with symptoms of typical angina and shortness of breath despite optimal medical therapy. In his relevant history, he had a coronary artery bypass graft (CABG) operation in 2009 in which his LIMA was anastomosed to left anterior descending (LAD) and ramus artery sequentially. Coronary angiography including selective imaging of LIMA demonstrated a fistula formation originating from the proximal portion of the LIMA and draining to PA. After successful closure of fistula with transcatheter coil embolization, the patient was discharged without any complication and symptom.

In conclusion, although LIMA to PA fistula is an infrequent clinical condition, it should be considered as a potential cause of persistent angina after CABG operation. Treatment options include conservative medical therapy, surgical ligation and endovascular interventions. The best therapy should be individualised for each patient in respect to patient’s symptoms, surgical compatibility and anatomy of fistula.

## Introduction

Left internal mammary artery (LIMA) is the most commonly used vessel as a bypass graft conduit to the left anterior descending artery (LAD) because of its long term patency. Although fistula between LIMA and pulmonary artery (PA) is a rare clinical condition, coronary artery bypass grafting (CABG) operation is a common cause of the acquired LIMA to PA fistulas. This clinical situation is important with regard to recurrent angina despite optimal medical therapy after CABG. Nowadays, endovascular interventions have been successfully applied for the treatment of fistula formations and are considered as the first line therapy options. In this paper, we report a LIMA to PA fistula and successfull treatment with transcatheter coil embolization.

## Case Report

**Figure 1:**
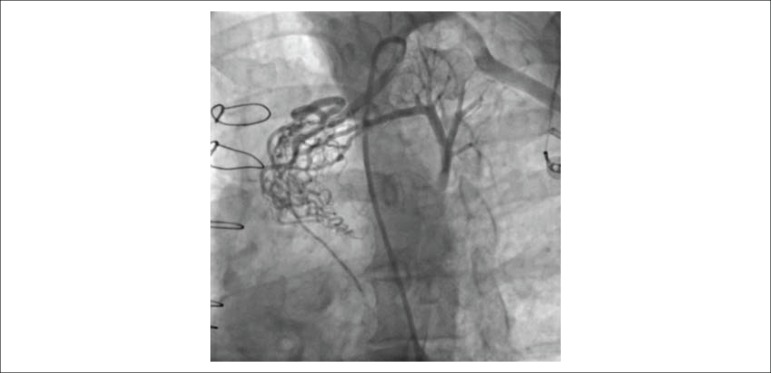
The angiographic view of the fistula formation between LIMA and pulmonary artery.

Fifty-three years old male patient who has a history of 2-vessel CABG in 2009 in which his LIMA was anastomosed to LAD and ramus artery sequentially admitted to our outpatient clinic with symptoms of typical angina and shortness of breath despite optimal medical therapy. Coronary angiography demonstrated 60% stenosis of proximal right coronary artery (RCA) with an FFR value 0.87, 90% stenosis of native ramus artery and a fistula formation from LIMA to PA before anastomosing to LAD and ramus artery (Figure 1). Computed tomography (CT) angiogram of coronary arteries and bypass grafts confirmed the diagnosis of fistula and its course. After consulting the patient with the Heart Team including cardiothoracic surgeons and anesthesiologists in sense of treatment strategy, we decided to completely revascularize the patient with stenting native Ramus artery and closing the fistula with transcatheter coil embolization in the same procedure. Our rationale for treating native ramus artery in the same procedure was aiming to block the sequential flow coming from LIMA by competing with it, which will result in augmentation of LIMA to LAD flow.

**Figure 2:**
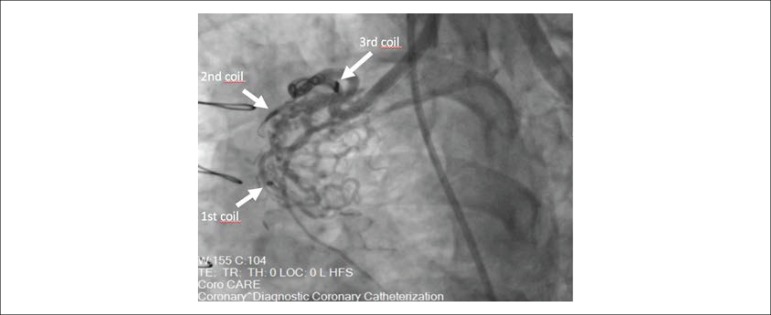
The angiographic view after first three coils which couldn’t completely occlude the fistula.

**Figure 3:**
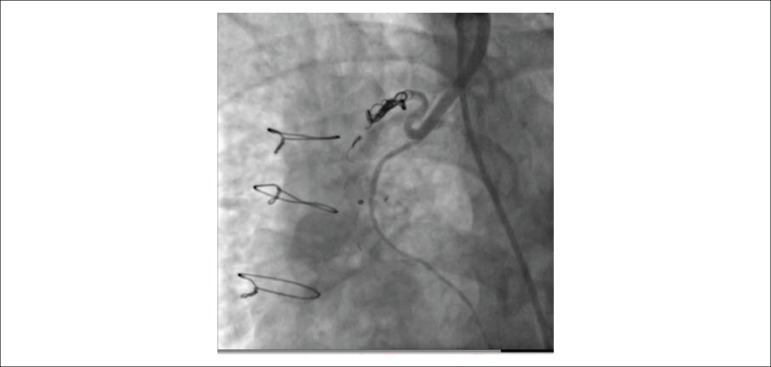
Total occlusion of the fistula after additional coil embolization.

After stenting ramus artery with a 3.0 x 15 mm drug-eluting stent, we carefully engaged the ostium of LIMA with an 8 French internal mammary artery (IMA) guiding catheter and advanced a 0.014’’ PT^2^ guidewire to the distal part of fistula. Next, 2.8 French CANTATA microcatheter *(Cook Medical, Bloomington, IN, USA)* was advanced to the midportion of fistula over the PT^2^ guidewire. Since the fistula was draining to PA with two major side branches which were dividing to multiple collaterals into their distal portions, our initial strategy was occluding the two major branches of fistula individually. However, the first coil (*11-2-2 HL spiral, Detach Coil System, Cook Medical, Bloomington, IN, USA)* was distally embolized inadvertently and the other two coils (*11-3-4 HL spiral* and *18-4-6 HL spiral)* for each side branch could not completely occlude the fistula (Figure 2). Hence, we changed our strategy and deployed an additional three larger coils (*18S-5-7 HL spiral, 18S-6-8 HL spiral, 18S-6-15 HL spiral)* to the main branch of fistula. After waiting for a few minutes, control angiography of LIMA demonstrated total occlusion of the fistula without any complication (Figure 3). The patient tolerated the procedure well and was discharged without any complication and symptom.

## Discussion

Fistula formation from LIMA to PA is an uncommon clinical entity. The first LIMA to PA fistula case was presented by Burchell and Clagett^1^ in 1947. Since then, such cases have been published very rarely. LIMA to PA fistulas can be congenital or acquired. The common cause of acquired form is CABG but it may also be secondary to trauma, infection, and neoplasm.^2-4^ Madu et al.^5^ reported the incidence of LIMA to PA fistula as 0.67% by means of reviewing 595 post-CABG angiograms and the meantime for development of such a fistula was about 5 years. A similar study, conducted by Guler et al.^6^ reviewed 537 post-CABG patients’ coronary angiogram and reported only 5 (0.93%) of them were having fistula formation originating from LIMA and draining to Pulmonary vasculature.

Several pathophysiologic mechanisms have been proposed for LIMA to PA fistula formation although the underlying cause is not fully understood. However, predisposing factors for the development of fistula include; the use of electro-cautery instead of ligation of the side branches while harvesting the LIMA,^7,8^ injury to the pleura and lung parenchyma^9^ that may cause direct contact between LIMA and pulmonary vasculature, operative site infection and inflammatory process leading to neovascularization.

The most common reported symptom is persistent angina despite optimal medical therapy. Coronary steal phenomenon and myocardial ischemia due to substantial shunting of blood from the LIMA to the PA is proposed as the underlying pathophysiological mechanism of angina.^5^ Nonetheless, patients may consult physicians with also dyspnea and other congestive heart failure symptoms. Continuous murmur can be heard on physical examination. Although it is very unusual, such a fistula formation can be complicated by myocardial infarction, congestive heart failure, pulmonary hypertension, arteritis, aneurysm or rupture.^6^

Selective angiography of LIMA has utmost importance to diagnose this fistula formation.^10^ Treatment options of LIMA-PA fistula include conservative medical therapy, surgical ligation and endovascular therapy options such as coil or vascular plug embolization and covered stent implantation. Asymptomatic patients with small fistulas and high-risk surgical patients who don’t have suitable anatomy for endovascular interventions may be followed up by conservative medical therapy; however, patients suffering from angina and congestive heart failure symptoms despite optimal medical therapy and patients having aneurysmal dilatation of fistula should be intervened either surgically or percutaneously. Currently, less invasive endovascular therapy options superseded surgical treatment which carries a significant morbidity and mortality risk.

Coil and vascular plug embolization techniques are the most commonly used endovascular therapy options for treating fistula formations. However, both techniques have pros and cons. Coil embolization is generally used for smaller fistulas^11^ and needs smaller catheters for delivery. Additionally, coils are low profile, easy to deliver and inexpensive devices when compared to vascular plugs. But, the Achilles' heel of coil embolization are high recanalization rates and distal embolization as we experienced in our case. On the other hand, vascular plug embolization is usually prefered for larger fistulas^11^ and provides more accurate placement with less distal embolization and recanalization rates. However, vascular plugs are more expensive than coils and require larger catheters for delivery (4 French at least). Also, delivery and deployment in tortuous vessels can be challenging in some cases due to rigid catheter-release wire set.^12^ Beyond the individual features of these devices, deciding the type of endovascular therapy depends on both the operator experience and anatomy of the fistula.

Having said that, since our case was suffering persistent angina despite optimal medical therapy and redo operation was constituting a significant surgical risk, we decided to treat the patient with endovascular intervention. In addition, the fistula formation was suitable for transcatheter coil embolization in terms of diameter and anatomy. Since the fistula was having a tortuous structure particularly in its proximal portion, we did not use vascular plug occluder in this case.

## Conclusion

LIMA to PA fistulas are rare conditions which can develop after bypass grafting and should be kept in mind as a cause of persistent angina. Selective angiography of the LIMA with careful evaluation of the images are substantial for proper diagnosis and treatment of this entity. Endovascular interventions are now considered first-line therapy options. Physician and institutional experience, as well as the anatomy and characteristics of fistula, are crucial while deciding the type of endovascular therapy.
